# Transition from methadone to subcutaneous buprenorphine depot in patients with opioid use disorder - a case report

**DOI:** 10.1192/j.eurpsy.2024.856

**Published:** 2024-08-27

**Authors:** M. Delic

**Affiliations:** Center for Treatment of Drug Addiction, University Psychiatric Clinic Ljubljana, Ljubljana, Slovenia

## Abstract

**Introduction:**

Opioid dependence is a complex condition that often requires long-term treatment and care. Methadone, a synthetic full opioid agonist, and buprenorphine, a partial agonist at the opioid receptor, are most commonly used for substitution therapy of opioid dependence and typically administered orally as a liquid and sublingual tablets. Transition from methadone to sublingual buprenorphine may precipitate withdrawal and is usually performed only in patients on low dose of methadone (<30-40 mg). Microdose induction is proposed as a possible solution to ease the transition to buprenorphine.

**Objectives:**

To present a rapid transition from methadone to sublingual buprenorphine and after that to buprenorphine depot.

**Methods:**

A case report of a patient who was switched from methadone 60 mg to sublingual buprenorphine 8 mg using microdosing and after that switched to buprenorphine depo 16 mg weekly.

**Results:**

Patient was successfully switched to sublingual buprenorphine and after that to buprenorphine depot. The transition was complited without withdrawal simptoms.

**Conclusions:**

This report supports the use of a microdose induction to initiate buprenorphine. Additionally, this approach may be significant for patients stabilized on high doses of methadone who may not be able to tolerate a traditional buprenorphine induction.

**Disclosure of Interest:**

None Declared

